# Parkinson Disease Psychosis: Update

**DOI:** 10.3233/BEN-129016

**Published:** 2013

**Authors:** J. H. Friedman

**Affiliations:** Movement Disorders Program, Butler Hospital, Department of Neurology, Alpert Medical School of Brown University, Providence, RIUSA

**Keywords:** Parkinson’s disease psychosis, hallucinations, delusions, atypical antipsychotic drugs, neuroleptics, parkinsonism, paranoia

## Abstract

Psychotic symptoms are common in drug treated patients with Parkinson's disease (PD). Visual hallucinations occur in about 30% and delusions, typically paranoid in nature, occur in about 5%. These problems, particularly the delusions, cause great distress for patient and caregivers, and are among the most important precipitants for nursing home placement. Psychotic symptoms carry a poor prognosis. They often herald dementia, and are associated with increased mortality. These symptoms often abate with medication reductions, but this may not be tolerated due to worsened motor function. Only clozapine has level A evidence to support its use in PD patients with psychosis (PDP), whether demented or not. While quetiapine has been recommended by the American Academy of Neurology for “consideration,” double blind placebo controlled trials have demonstrated safety but not efficacy. Other antipsychotic drugs have been reported to worsen motor function and data on the effectiveness of cholinesterase inhibitors is limited. PDP remains a serious problem with limited treatment options.

## 1. Introduction

Until recently, PD, defined clinically by its motor aspects, has become more accurately seen as a neurobehavioral disorder [1,2] in which the behavioral aspects are generally the major determinants of quality of life [3,4], nursing home placement [5,6], and caregiver stress [7,8]. While many of these behavioral problems, such as depression and dementia, are clearly intrinsic to the disease, some, such as impulse control disorders, are clearly related to its treatment [9,10], and others, like the psychotic symptoms, represent an interplay between the two [11].

Psychotic symptoms were known to have occurred with post-encephalitic parkinsonism (PEP) in the early twentieth century [12,13] and for a time, some authorities believed that idiopathic PD and PEP were related entities [14] despite the observation that the two were pathologically quite distinct. Psychotic symptoms were rarely described in idiopathic PD until L-Dopa was introduced [15]. Because of the clinical and pathological overlap between Dementia with Lewy Bodies (DLB) and PD [16] experts currently argue as to whether these are two separate disorders or not. While DLB had initially been considered "the Lewy body variant of Alzheimer's disease," it is now, more appropriately thought of as a variant of PD. Since one of the cardinal features of DLB is visual hallucinations (VH) [16,17], it is reasonable to wonder whether some psychotic symptoms seen in treated PD patients may be intrinsic to the disease and not related to medications [11]. One report on patients with advanced but untreated PD indicates that psychotic symptoms may occur without dementia [18]. It is also important to keep in mind that PD may occur in people with primary psychotic disorders [19,20]. However, the psychotic symptoms of PDP are generally quite different than those which occur in primary psychiatric disorders.

### *1.1. Clinical aspects*

Psychotic symptoms in PD are generally quite stereotyped [21–26]. Hallucinations are clearly the most common, and have been subdivided into "minor" and non-minor [11]. "Minor hallucinations" refer to three distinct phenomena. One is the transient visual

ISSN 0953-4180/13/$27.50 © 2013 – IOS Press and the authors. All rights reserved hallucination that occurs in the peripheral field. Patients describe seeing a shadow or an animal, like a dog, in either peripheral field and often turn to see what it is, only to have it disappear. These are not seen distinctly and do not occur in the central field. Oftentimes patients describe seeing dots of light in the peripheral field and believe these are due to reflections off their eyeglasses. The second phenomenon is not an actual hallucination although called "presence hallucination." These are feelings that there is a person or animal behind, or nearby, but not seen. The patient will turn to see who or what is there, only to find nothing. These feelings are not paranoid and are not associated with a concern about being attacked from a blinded area. The third type of "minor hallucination" is the illusion, almost always visual. Real objects are seen transformed into other shapes. A fire hydrant at a distance becomes a man walking a dog. Pictures of flowers become faces, etc. Visual illusions are common in normal people but in PD these may become excessive and are recognized by the patient as being clearly abnormal.

A proposed set of criteria for defining PDP arose from a consensus conference [11]: recurrent or continuous symptoms for one continuous month; delusions, hallucinations or "minor" hallucinations; diagnosis of idiopathic PD (not DLB); no other explanation found. Subcategories were then suggested, in accord with DSM methodology: with or without PD medications; with or without dementia.

The prevalence of psychotic symptoms varies with the population. In a study of three community based practices which focused on PD in the U.S., 26% of 250 non-demented patients had psychotic symptoms. 52% of these patients had hallucinations or delusions, the rest having minor symptoms. Cumulative incidence is much higher [27]. Their presence was associated with poorer quality of life [24]. In general, hallucinations occur in about 15–30% of drug treated patients [28]. Visual are the most common type of hallucination, followed by auditory, then by those affecting the other senses, tactile, olfactory, gustatory [29]. Visual hallucinations are typically not frightening, and are usually of people or animals, although inanimate objects may also be hallucinated. They generally appear when the patient is in a low stimulus environment, more often in the evening or at night [22]. They may appear for seconds or minutes. The hallucinations often ignore the patient, but not always. Interestingly the hallucinations tend to be fairly stereotyped. Patients tend to see the same group of people, dressed in the same manner each time they hallucinate. They will see a parade outside every day, or the same friendly dog. When approached, the hallucinations always disappear. The hallucinations may be in black and white, or color. The people may look normal, mildly abnormal, or indistinct. They may wear odd outfits. Some patients find the hallucinations mildly entertaining or amusing, but most find them bothersome, mostly because they indicate a problem with their mind, not because they are frightening. A common, very annoying visual hallucination is seeing bugs on the floor or wall. The auditory hallucinations tend to be different than the visual. They are usually indistinct sounds, like a radio playing in another room, a band performing in a house across the street, people talking outside the room. Occasionally the visual hallucinations will carry on conversations with the patient. Unlike the classic symptom of schizophrenia in which voices talk about the patient, these conversations involve the patient and are not denigrating or threatening. The other hallucinations also are episodic, smelling perfume, ammonia, picking at insects on the skin, etc.

Delusions are considerably less common and affect about 5% of drug treated patients [29]. The delusions are usually very distressing as they tend to be paranoid in nature, with patients thinking that their spouses are having romantic affairs, that people are planning on harming them, that they are about to be placed in nursing homes, that their house is being sold or their bank account is being raided [21]. Grandiose and bizarre delusions, common in primary psychotic disorders, are virtually never seen. "Secondary delusions" are false, irrational beliefs based on hallucinations, and may develop in PDP. Thus a patient who feels insects crawling on his skin, although he can see none present, will believe that he really does have insects on his skin and spray insect repellent on himself, which he finds useless.

The differential diagnosis of PDP includes delirium, Bonnet's syndrome, dream-related behavior, such as Rapid Eye Movement (REM) sleep behavior disorder, vivid dreams, hypnogogic and hypnopompic hallucinations. Delirium differs from PDP due to decreased attention, variable level of consciousness, disorientation and illogical thinking.

The only uniformly accepted risk factor for psychotic symptoms is dementia [29], supporting the association between PDP and DLB. But psychotic symptoms occur in non-dements as well, even when patients have been followed for many years after onset (personal observation). Other risk factors reported include reduced vision [28] for VH, age, duration of illness, REM sleep behavior disorder [30], dopamine agonists, axial rigidity subtype [31–33]. One study of 48 subjects found that non-demented hallucinators differed from non-hallucinators by having reduced executive dysfunction including verbal learning-immediate recall and verbal fluency tasks [34]. The same group expanded this observation in a longitudinal study which demonstrated that verbal fluency impairment predicted the development of hallucinations [35]. Other reports have also described an association between impairment of executive function and visual hallucinations [36].

Although dementia is a risk factor for psychotic symptoms, it is also true that hallucinations are a significant risk factor for dementia, often a herald symptom [29] PD dementia has long been associated with a cholinergic deficit, and an indirect measure of cholinergic function in the motor cortex, short latency afferent inhibition, has been employed to show that PD patients with VH had more dysfunction than non-hallucinators [37]. This complements the association noted above [34] of VH with executive dysfunction in non-demented PD patients, suggesting the hypothesis that VH are a "herald symptom" of PD dementia.

The pathophysiology of PDP is unclear. There is far more written about hallucinations than delusions in PDP. Typically hallucinations precede the development of delusions and delusions usually develop in people who already have hallucinations [21]. One hypothesis suggested that hallucinations were actually partial syndromes of RBD [38]. RBD does appear to be a risk factor for the development of PDP [39]. Perhaps the most interesting study on hallucinations was one in which patients who hallucinated nightly at home were brought to a hospital where they were given extra large doses of L-Dopa, in order to determine an association between serum levels and the occurrence of hallucinations. None of the subjects had hallucinations, underscoring the importance of mental state in the development of this syndrome [40].

A series of 63 PD patients followed to autopsy described Lewy body pathology in the amygdala as a unifying abnormality distinguishing PD patients with hallucinations from those without [41], however, this was not confirmed in another study of 94 PD patients [39]. Other temporal pathology has also been reported [39, 42], as well as Lewy bodies in the frontal, and temporal cortices [43]. Data conflict on the importance of Lewy body density in the parietal cortex [39,40]. Hallucinations in non-PD parkinsonian syndromes are rare [42, 44,45], although this may partly be explained by the lesser use of PD medications in these other disorders. Visual acuity has been reported to be less in hallucinators than in non-hallucinators [28], but this too has not always been found [39]. Visual processing impairment has been implicated [39,46,47]. The PDP patients were not thought to suffer from Bonnet's syndrome in these series.

One potential hypothesis to explain the occurrence of PDP in some but not all subjects is a genetic predisposition. There is pilot data to suggest such a possibility [48], however the cumulative incidence of psychotic symptoms is so high, over 50%, that it seems unlikely that a simple genetic explanation will be found. Genetic risk factors have been evaluated, with no clear association found. Examined genes include MAPT, SNCA-REP1, APOE subtypes, ACE II, 5HT2A receptor, dopamine transporter gene polymorphisms, cholecystokinin promoter polymorphisms and COMT [49]. Since psychotic symptoms are associated with dementia, it is likely that genetic contributors to dementia in PD will also be contributory to psychotic symptoms.

Positron emission tomography (PET) studies on PD patients with visual hallucinations have reported reduced metabolic activity in the occipital-parietal-temporal regions, consistent with alterations in visual association areas [50]. FMRI has revealed hypometabolism in response to visual stimulation in visual association cortex in PD patients who had visual hallucinations [51]. Similar results have been described in dementia with Lewy bodies [52,53]. The role of neurotransmitter changes in VH and other psychotic symptoms is unclear. Aside from the data implicating cholinergic deficits cited above, the provocation of psychotic symptoms with L-Dopa and dopamine agonistis, there is also data implicating serotonin excess in the temporal cortex [54]. Gallagher et al. [39] have hypothesized a model, based on a survey, to explain visual hallucinations, which involves a heavier pathological burden of disease, sleep disturbances, which include an increase in REM sleep behavior disorder and disturbed sleep cycles, worse cognition, worse motor dysfunction, and more autonomic dysfunction. However, this does not account for the relationship of drugs, and the common occurrence of hallucinations early in the disease on dopamine agonists, when patients have mild signs and symptoms of the disease. Furthermore, this model does not lead to an explanation for the other types of hallucinations or the later development of delusions.

Although psychotic symptoms are treatable, they are associated with a poor outcome. Treating the symptoms early is associated with a better outcome [55].

One small prospective study of nursing home residents with PDP found 100% mortality after two years [56]. In the PSYCLOPS study [57], the first multicenter trial of clozapine for PDP, 10% of the subjects died within a few months of the study's completion, despite treatment. VH are often a herald symptom for dementia [29]. And, as noted above, caregiver stress is markedly increased in PDP patients, leading to increased rates of nursing home placement.

### *1.2. Management*

Algorithms for treating PDP have not changed significantly from the first recognition that clozapine was useful and well tolerated [58]. Since psychotic symptoms may be triggered by somatic disorders, particularly infection, these must be assessed as potential contributory factors. Oftentimes these are occult, with little outward evidence of pneumonia, bronchitis or urinary tract infections. All psychoactive medications must also be considered. It is not uncommon for PD patients to be taking sedatives, anxiolytics, antidepressants and anticholinergics for bladder control. When possible these agents should be reduced and eliminated. There is no data to guide the treating physician on how to do this, but common practice is to reduce and discontinue as many as possible. Obviously these choices must be dictated by the circumstances. In the case of anticholinergic drugs for bladder control, it appears that the newer agents have fewer neurological side effects than the older ones so that a bladder medication might be replaceable by something equally effective with fewer mental effects. Once the non-PD medications are at their minimum then the anti-PD medications may need to be reduced. Clearly such a reduction will, at some point, result in worsened motor function. The patient and family must be prepared for this. Each medication's effect must be weighed with consideration for the particular patient. Generally anticholinergics and amantadine are the first to be removed. While I advocate tapering and stopping one medication at a time, following the general approach to toxicity in the epileptic patient, there is no data to support this. After this, monoamine oxidase b-inhibitors (MAOb) may be stopped, although these drugs are suicide inhibitors and may take weeks to actually wash out of the brain. There is little to be gained by stopping the catechol-o-methyltransferase inhibitors (COMT-I) as they do not work directly in the brain, producing effects by increasing serum levels of L-Dopa. Dopamine agonists are next reduced and stopped, with COMT and L-Dopa reduction held for last. Since reducing these medications will often result in worsened motor function, the patient must either tolerate the improvement in psychosis as a trade off or may need to have L-Dopa increased.

Prior to the introduction of clozapine there was no effective treatment for PDP. The "drug holiday," which had evolved from a treatment regimen for severe clinical fluctuations, was also used for psychotic symptoms but was not effective [59]. This approach was based on the assumption that the drug response problems were related to dopamine receptor super-sensitivity and that by stopping their stimulation for 10–14 days their normal sensitivity would be restored and lower doses of L-Dopa could be used without side effects. This method was abandoned as ineffective. Since the treatment of the PD motor symptoms was limited by these psychotic symptoms that could not be avoided, the next approach to its treatment involved using an antipsychotic, thus treating medication side effects with yet more medication.

Many open label reports involving hundreds of patients at multiple centers attested to clozapine's efficacy without impairment of motor function [60–66], with doses that were extremely small compared to the doses used in treating schizophrenia. Two double blind, placebo controlled multi-centered trials were performed of low dose clozapine for PDP [57,67]. Although these studies were performed independently, one in the U.S. and one in Europe, the two used almost identical protocols. The results of the two studies were quite similar. Clozapine, at very low doses, 25 mg/d mean dose in the US and 35 mg/d in the European study, was effective, with dramatic reductions of psychosis, and good tolerance, without significant change in motor function, with a trend towards better motor function. The mean tremor scores had improved with clozapine. For comparison, the doses recommend for schizophrenia are 300–900 mg/d. In the U.S. study, the single most commonly used dose was 6.25 mg/d. The occurrence of agranuloctyosis, which is not a dose related phenomenon, did not appear increased in PD patients, but does occur, even at very low doses. However, long term use at the low doses used in PDP do not seem to induce the metabolic syndrome, as commonly seen with the higher doses used in schizophrenia [68, 69]. Other reports confirmed the benefit of clozapine, at tolerable doses, for tremor in PD [70–73], even in patients who were refractory to the usual anti-PD medications.

Other antipsychotic medications have been tried in PDP. After a single open label study demonstrating a good effect of olanzapine in PDP [74], rivaling that reported in the open label reports for clozapine, four double blind controlled trials were launched [75–77]. Unfortunately, olanzapine produced similar results in all studies: the drug was ineffective for the psychosis and worsened Parkinson motor features. Open label trials, published after the double blind trials began reported results similar to the double blind, controlled trials [78]Results for risperidone [79–82], ziprasidone [83,84] and aripiprazole [85–87] have all been open label and conflicting in their outcomes, with some demonstrating worsened parkinsonism [81,83, 88,89] and others reporting good tolerance [79,80,90–92], without worsening of motor function. Some reports have found "non-inferiority" of drugs compared to clozapine [93–95]. The drugs have generally been reported helpful for the psychosis, although not uniformly. Aripiprazole is the most interesting of the "atypical" antipsychotics, in that it alone has both dopamine receptor blocking as well as dopamine agonist properties, thus theoretically making it the ideal drug for PDP. However, small, open label, trials [85–87], including a prospective study [86] revealed worsened motor function. In addition, risperidone, olanzapine, ziprasidone and aripiprazole have frequently been associated with dose-dependent neuroleptic induced parkinsonism in otherwise normal psychotic patients (personal observation). Open label studies showed quetiapine to be well tolerated and an effective antipsychotic for PDP [96–98] and in comparison trial [95] to be non-inferior to clozapine. Unfortunately, three double blind controlled trials did not find efficacy [99–101], although all confirmed that the drug was free of motor worsening. One small DBPCT study [102] did find efficacy and tolerance but the numbers are too small to be persuasive. Finally, melperone, which was reported to be effective and well tolerated in a study in Italy [103], was the subject of a multi-center DBPCT in the U.S. which found the drug to be ineffective against psychosis although well tolerated, without a decline in motor function [104].

Anti-psychotic drugs released in the U.S. since aripiprazole (iloperidone, asenapine, lurasidone) have not yet been the subject of published reports for their effects in PDP. Another approach comes from the DLB literature, where the cholinesterase inhibitors have had some beneficial effects on hallucinations and delusions, with good tolerance. Since the clinical manifestations of DLB and PDP seem very close, if not identical, other than the obvious difference that many of the PDP patients are not demented, it would seem reasonable that they may also be helpful in PDP. The effects of CEI in PDP have been the subject of only a few open label trials [105–109], showing good tolerance and some benefit. One problem with such an approach is the long lag time between drug initiation and clinical response. Usually these drugs are given at a low dose for four weeks before being increased. The anti-psychotics often work within days. There are two reports of ondansetron, a 5HT3 antagonist [110,111] being effective, and trials of pimavanserin, a 5HT2-A antagonist not having statistically beneficial results [112,113].

An oft overlooked approach with refractory severe PDP cases is electroconvulsive therapy (ECT). However, this approach is based on case report material only [114–117]. It should be noted that many case reports state that ECT, when used in PD, usually for depression, often improved motor function for days to weeks.

Only clozapine has A-level support for its use in treating PDP. The Movement Disorders Society review committee on evidence-based medicine notes that as of 2011 only clozapine had convincing evidence of benefit. In a 2007 review by the American Academy of Neurology's task force on the treatment of PD, clozapine and quetiapine were recommended for "consideration" only [118], the former based on its potential for agranulocytosis and the latter based on the lack of DBPCT data to support efficacy. This committee also recommended quetiapine for "consideration."

PDP should be considered an unmet need in PD. Although clozapine has been found to be effective, it is sometimes a difficult drug to use because of the blood monitoring requirement. A small percentage of patients may suffer a drop in granulocytes that precludes their taking the drug. While the problems associated with the metabolic syndrome do not occur with the low doses of clozapine required for PDP, excess sedation and orthostatic hypotension may occur even at very low doses. In the United States, psychiatrists are usually extremely reluctant to prescribe clozapine, considering it too unsafe. No other antipsychotic drug has been found to be effective and all but quetiapine and melperone have either been found to worsen motor function or have not yet been the subject of even case reports concerning PD.
